# Membranous Nephropathy With Monoclonal IgM Lambda Deposits in a Patient With IgM Monoclonal Gammopathy: A Case Report

**DOI:** 10.3389/fmed.2021.608741

**Published:** 2021-05-25

**Authors:** Go Hirose, Takahiro Uchida, Aki Kojima, Kentaro Sugisaki, Muneharu Yamada, Yoshihiro Nagase, Takashi Takaki, Kiyotaka Nagahama, Takashi Oda

**Affiliations:** ^1^Department of Nephrology and Blood Purification, Kidney Disease Center, Tokyo Medical University Hachioji Medical Center, Hachioji, Tokyo, Japan; ^2^Division of Electron Microscopy, Showa University School of Medicine, Tokyo, Japan; ^3^Department of Pathology, Kyorin University School of Medicine, Tokyo, Japan

**Keywords:** membranous nephropathy, immunoglobulin M, monoclonal gammopathy of renal significance (MGRS), rituximab, thrombospondin-type-1-domain-containing-7A

## Abstract

We report a case of membranous nephropathy with monoclonal immunoglobulin (Ig)M lambda deposits in a patient with IgM monoclonal gammopathy, in whom histological changes were observed on repeat renal biopsy. A 72-year-old Japanese woman was referred to our hospital because of massive proteinuria. A prominent increase in monoclonal IgM lambda level was identified, and she was diagnosed as having IgM monoclonal gammopathy of undetermined significance. Renal biopsy showed glomerular subepithelial electron-dense deposits that were found to be granular deposits of IgM lambda but not kappa or IgG by immunofluorescence staining, resulting in a diagnosis of membranous nephropathy with monoclonal IgM deposits. The second biopsy, which was performed 2 years later because of exacerbation of her nephrotic syndrome, demonstrated less immunofluorescence staining of IgM, and dominant IgG2 deposition without light chain restriction. Interestingly, immunostaining for thrombospondin-type-1-domain-containing-7A was positive in both renal biopsy tissues, although the second biopsy showed clearly stronger immunoreactivity. The effect of steroid therapy was limited; however, rituximab treatment improved both the hematological and renal abnormalities. Solitary deposition of IgM in membranous nephropathy is a quite rare condition. To our knowledge, this is the first case of monoclonal gammopathy of renal significance presenting as membranous nephropathy with monoclonal IgM deposits, in which chronological immunohistochemical changes were observed and rituximab therapy was effective.

## Introduction

Membranous nephropathy (MN) is a type of glomerular disease that causes the deposition of immune complexes along the subepithelial region of the glomerular basement membrane, and often clinically results in nephrotic syndrome in adults. MN is broadly separated into the following two categories: idiopathic MN, in which patients do not have an underlying disease, and secondary MN, which is associated with a causative systemic disease, such as infection, autoimmune disease, or malignancy. Podocyte phospholipase A_2_ receptor (PLA_2_R) was the first reported target antigen for the immune complexes observed in patients with idiopathic MN (1). Subsequently, another target antigen, thrombospondin-type-1-domain-containing-7A (THSD7A) (2), was identified, and more recently, several other antigens have been proposed (3). Importantly, these antigens have been suggested to be associated with the etiology of patients with MN (3, 4), and the expression of THSD7A may also be associated with malignancy (5, 6).

In 2012, the term monoclonal gammopathy of renal significance (MGRS) was proposed to distinguish monoclonal gammopathy resulting in the development of renal disease from those without renal involvement (7). A variety of renal diseases have now been described to be associated with MGRS, and atypical MN accompanied with monoclonal immunoglobulin (Ig) deposition is also considered pathologically to be a type of MGRS (8, 9). This form of glomerulopathy is also referred to as non-organized and non-Randall-type monoclonal Ig deposition disease (MIDD) associated with membranous features (10), and proliferative glomerulonephritis with monoclonal IgG deposits with predominant membranous features (11, 12). However, the terminology for this condition has not been fully established, and the target antigen in this form of MN also remains unclear.

We herein report a case of a patient with MGRS presenting as MN with monoclonal IgM lambda deposits accompanied by IgM lambda monoclonal gammopathy, in whom renal biopsies were performed 2 years apart. In this patient, isolated granular deposition of IgM lambda along the glomerular capillary walls was observed in the first renal biopsy, whereas in the second biopsy, immunofluorescence (IF) staining of fresh frozen tissue sections showed that IgM deposits appeared to have diminished, and polyclonal IgG deposition had become dominant. Furthermore, immunostaining for THSD7A demonstrated positive glomerular staining in both renal biopsy tissues, and the staining pattern changed from focal and segmental weak granular staining in the first biopsy to diffuse and global strong granular staining in the second biopsy. Although steroid therapy was not effective, rituximab treatment improved both the hematological and renal abnormalities.

## Case Presentation

A 72-year-old Japanese woman, who was married and was unemployed, was found to have proteinuria at her local clinic, and was referred to our hospital for further examination. Her presenting symptoms were peripheral edema in both lower legs and a recent 2 kg increase in body weight. Her vital signs were normal. Her medical history included well-controlled dyslipidemia and osteoporosis. She had no family history of renal disease.

Her urinalysis showed a protein level of 4.6 g/day but no hematuria (1–4 red blood cells/high-power field). The results of her blood analyses were as follows: white blood cell count, 4.9 × 10^3^/μL; hemoglobin level, 13.8 g/dL; platelet count, 32.9 × 10^4^/μL, serum creatinine, 0.48 mg/dL; blood urea nitrogen, 13.5 mg/dL; total protein/albumin (TP/Alb), 7.6/3.1 g/dL; and IgG, IgA, and IgM, 474, 79, and 1,940 mg/dL, respectively. Antinuclear antibody titer was 1:80, which was slightly positive, but hypocomplementemia was absent. C-reactive protein and cryoglobulin were negative, and soluble IL-2 receptor level was 338 U/mL (reference range, <486 U/mL). Viral antibodies for hepatitis B and C were negative.

A prominent increase in the level of IgM and a decrease in the levels of other Igs prompted us to perform a detailed examination. A computed tomography scan did not display any bone lesions or lymphadenopathy. Serum protein electrophoresis showed a peak for monoclonal IgM lambda, and urine protein electrophoresis detected lambda-type Bence Jones protein. Concentrations of serum light-chain kappa and lambda were 12.9 and 62.7 mg/mL, respectively, and the free light-chain ratio was slightly low (0.21; reference range, 0.26–1.65). Bone marrow aspiration showed hypoplastic bone marrow with <10% plasma cells, which met the diagnostic criteria for monoclonal gammopathy of undetermined significance (data not shown).

**Figure 1:**
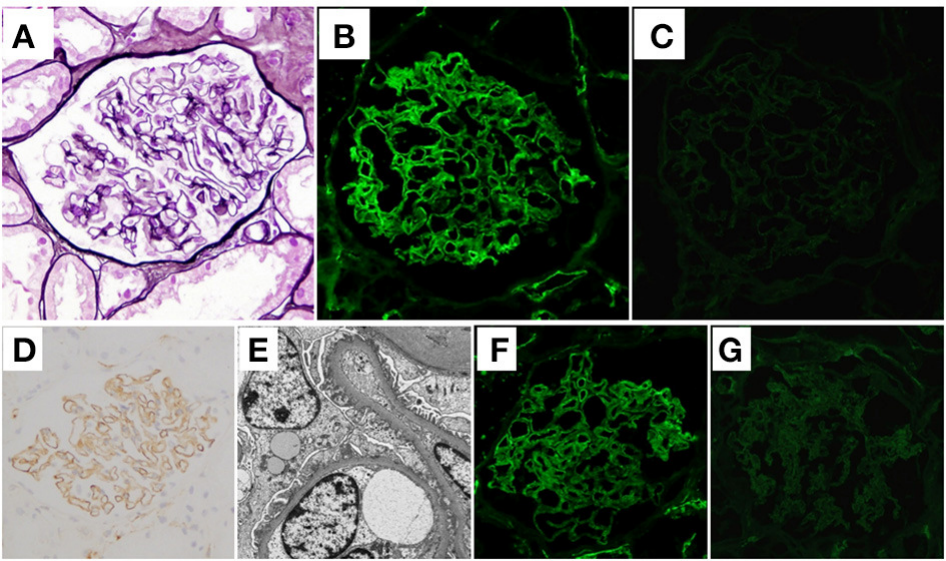
Histological features of the first renal biopsy. **(A)** Neither proliferative changes nor thickening of glomerular capillary walls was observed in the sections (periodic acid-methenamine-silver stain) on light microscopy. **(B)** Granular deposition of immunoglobulin (Ig)M on glomerular capillary walls was shown on immunofluorescence (IF) staining. **(C)** On the other hand, IF staining for IgG was negative. **(D)** Deposition of IgM on glomerular capillary walls was further confirmed by immunoperoxidase staining on formalin-fixed, paraffin-embedded tissue sections using an automatic staining machine by a clinical laboratory testing company (SRL, Inc., Tokyo, Japan). **(E)** Electron microscopy sections identified electron-dense subepithelial deposits. The effacement of podocyte foot processes is also shown. IF light-chain staining showed that whereas there was strong deposition of lambda chains on the glomerular capillary walls **(F)**, there was no deposition of kappa chains **(G)**.

A renal biopsy was performed to histologically analyze the cause of the massive proteinuria. Sections of 11 glomeruli were analyzed by light microscopy (LM), which showed that 2 of the glomeruli were globally sclerotic. There were no proliferative changes, and the glomerular capillary walls were not thickened (Figure 1A). There were minimal tubulointerstitial changes. Routine IF staining on frozen tissue sections showed granular 2+ deposition of IgM (Figure 1B) on the capillary walls, without deposition of IgG (Figure 1C), IgA, complement C1q, or C3 (data not shown). Immunoperoxidase staining for IgM on formalin-fixed, paraffin-embedded tissue (FFPE) sections were performed using an automatic staining machine by a clinical laboratory testing company (SRL, Inc., Tokyo, Japan). The stained slides further confirmed the deposition of IgM on the glomerular capillary walls (Figure 1D). Subepithelial electron-dense deposits were found on electron microscopy, but spike formation of the glomerular basement membrane was obscure (Figure 1E). Although stage I MN was suggested, IF staining for light chains demonstrated the deposition of only lambda chains but not kappa chains (Figures 1F,G). To rule out the existence of masked Ig deposits, we further performed IF staining for IgG, IgA, IgM, and light chains on FFPE sections after protease digestion as previously described (13), which also showed the granular deposition of IgM and lambda chains on the capillary walls, but with no deposition of IgG or kappa chains (Supplementary Figure 1).

**Figure 2:**
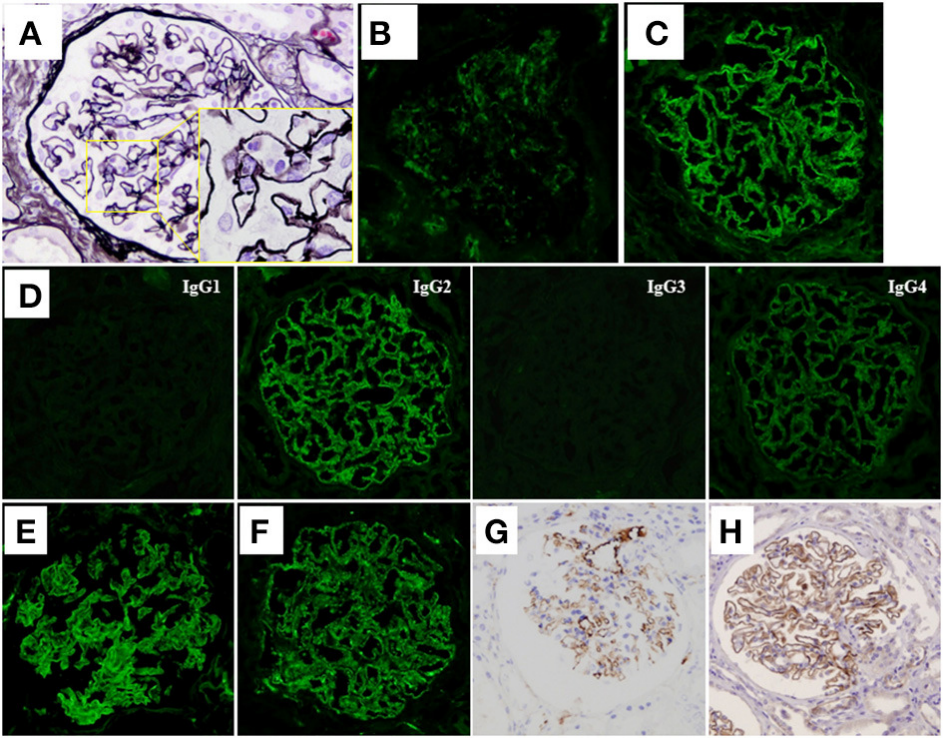
Histological features of the second renal biopsy. **(A)** Thickening of the glomerular capillary walls accompanied with spike formation and a bubbly appearance (see inset) was observed in the sections (periodic acid-methenamine-silver stain) on light microscopy. **(B)** IF staining for IgM demonstrated weak reactivity on the glomerular capillary walls. **(C)** IF staining for IgG was extensively positive on the glomerular capillary walls. **(D)** IF staining for IgG subclasses demonstrated dominant deposition of IgG2. Deposition of IgG4 was faintly observed. IF staining for light chains showed extensive deposition of both lambda chains **(E)** and kappa chains **(F)**. **(G)** Unlike IF staining, immunoperoxidase staining for IgM of formalin-fixed, paraffin-embedded tissue (FFPE) sections after antigen retrieval, using an automatic staining machine by a clinical laboratory testing company (SRL, Inc.) showed strong IgM deposition on the glomerular capillary walls. **(H)** Immunoperoxidase staining for thrombospondin-type-1-domain-containing-7A on FFPE sections showed diffuse global-positive staining on the glomerular capillary walls.

According to these findings, a diagnosis of MN with monoclonal IgM lambda deposits was made, and the patient was at first conservatively treated with an angiotensin II type 1 receptor blocker (ARB), because her clinical symptoms were mild with minor abnormalities in laboratory data, such as serum albumin and creatinine levels, and she did not wish to undergo aggressive treatment. However, although the level of IgM remained unchanged, her level of proteinuria increased, and she developed full nephrotic syndrome (urinary protein excretion, 11.0 g/day and TP/Alb, 5.2/1.1 g/dL) about 2 years after the first biopsy. Her renal function also worsened, and her serum creatinine level was increased to 1.47 mg/dL [estimated glomerular filtration rate (eGFR), 27.3 mL/min/1.73 m^2^]. At that point, her serum proteins were re-evaluated for the presence of M-protein by immunofixation, which again showed only the monoclonal IgM lambda protein, thereby ruling out the presence of another paraprotein. A second renal biopsy was therefore performed to re-evaluate her renal histology about 2 years after the first biopsy. LM observation of sections showed that 2 of the 28 glomeruli analyzed were obsolescent, and interstitial fibrosis and tubular atrophy were moderate. Thickening of the glomerular capillary walls accompanied by spike formation and a bubbly appearance was also observed, suggesting disease progression of MN (Figure 2A). IF staining of frozen tissue sections demonstrated weakened IgM staining (Figure 2B) and augmented IgG staining (Figure 2C), in which IgG2 was dominant with weaker deposition of IgG4 (Figure 2D). Light chain restriction was not observed by IF staining this time, and both lambda and kappa light-chain deposits were observed at almost equal levels (Figures 2E,F). Thus, in the second renal biopsy, routine IF staining on a frozen tissue section showed weak IgM staining in glomeruli; however, immunoperoxidase staining for IgM on a FFPE section after antigen retrieval showed strong IgM deposition on the glomerular capillary walls (Figure 2G).

**Figure 3:**
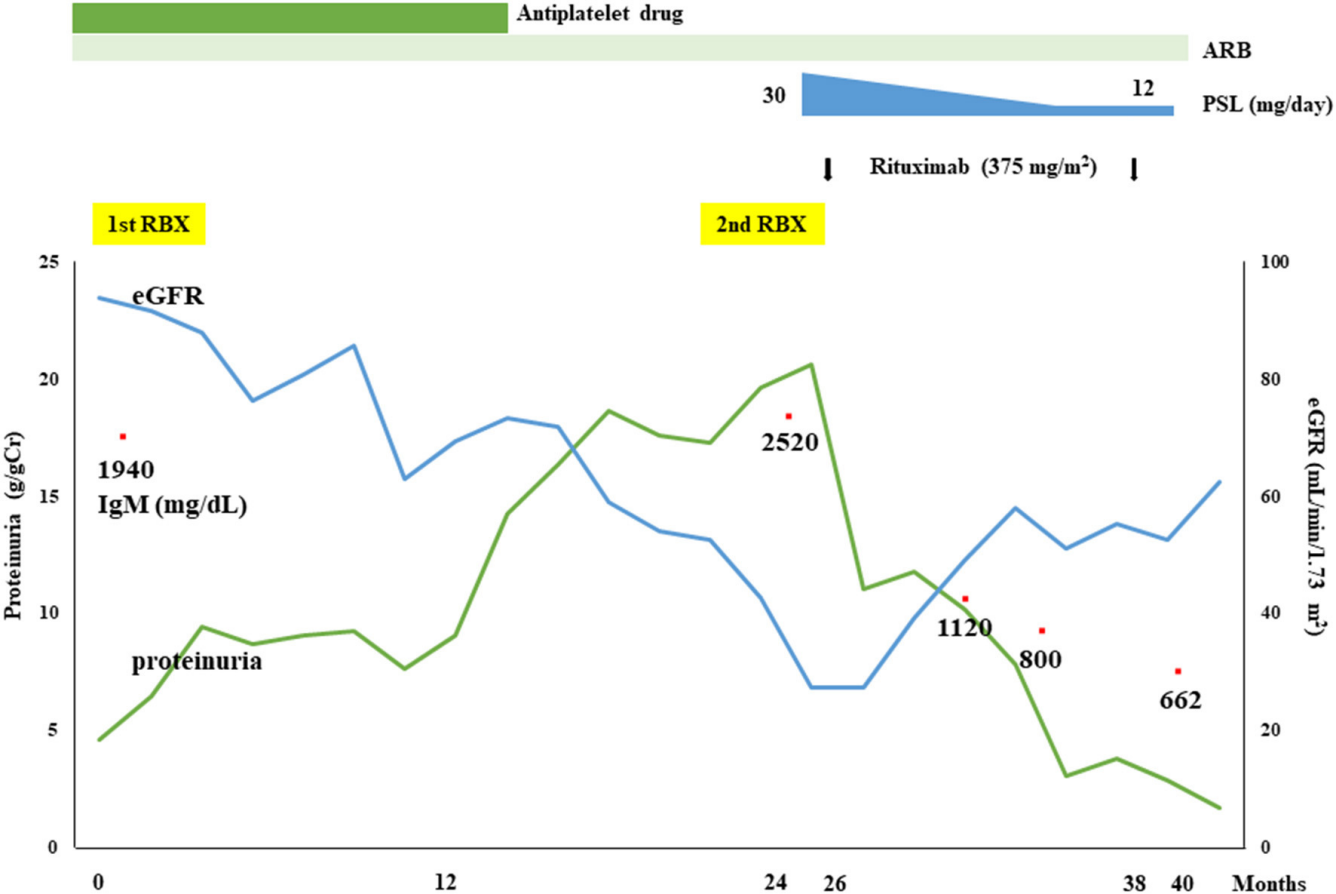
Clinical course of the present patient. Although the therapeutic effects of an angiotensin II type 1 receptor blocker (ARB) and a steroid (prednisolone; PSL) were insufficient, a substantial reduction in proteinuria and improvement of renal function as well as hematological responses were achieved by rituximab treatment.

Although IF staining of IgG was augmented in the renal tissue of the second renal biopsy, serum anti-PLA_2_R antibody level measured by enzyme-linked immunosorbent assay, and IF staining for PLA_2_R on FFPE sections were both negative (Supplementary Figure 2). Immunoperoxidase staining for neural epidermal growth factor-like 1 protein (NELL-1) on FFPE sections was also negative (Supplementary Figure 2), whereas that for THSD7A on FFPE sections showed a diffuse positive staining pattern on the glomerular capillary walls (Figure 2H). Immunoperoxidase staining for THSD7A on FFPE sections of the first renal biopsy was therefore performed and the staining result was compared with that of the second renal biopsy. Compared to the second renal biopsy, which showed diffuse and global strong staining, the staining result of the first renal biopsy showed focal and segmental granular immunoreactivity on the glomerular capillary walls (Supplementary Figure 3). THSD7A was negative in cells of the bone marrow by immunoperoxidase staining of FFPE sections (Supplementary Figure 3). Immunoperoxidase staining for amyloid P, a recently identified marker of membranous-like glomerulopathy with masked IgG deposits (14), was negative in the FFPE sections of both renal biopsy tissues (Supplementary Figure 2). Because of her severe nephrotic syndrome, the patient started steroid therapy; however, the effects were limited both for her renal and hematological abnormalities; i.e., her heavy proteinuria persisted and her serum IgM level remained high. On the contrary, 2 injections of rituximab (375 mg/m^2^ per injection) substantially improved both abnormalities; her renal function (eGFR), urinary protein level, and serum IgM level were maintained at more than 60 mL/min/1.73 m^2^, <1 g/day, and <1,000 mg/dL, respectively, after 2 rituximab injections. Her clinical course is shown in Figure 3.

## Discussion

MN is the most common cause of nephrotic syndrome in adults, and IgG4 deposition without light chain restriction is usually observed in the glomerular immune deposits of patients with idiopathic MN. However, in the present patient, the first renal biopsy showed isolated glomerular IgM lambda deposits, resulting in a diagnosis of MN with monoclonal IgM deposits. Larsen et al. (14) recently reported that serum amyloid P was a sensitive and specific marker of membranous-like glomerulopathy with masked IgG kappa deposits; however, immunostaining for amyloid P was negative in renal biopsy tissues of the present patient. In addition, IF staining using FFPE sections also showed deposition of IgM and lambda chains on the capillary walls, but with no deposition of IgG or kappa chains, which further supported our diagnosis. Kitazawa et al. (15) reviewed 18 reported cases and their own case of so-called MIDD associated with membranous features, that is, MN with monoclonal Ig deposits. The glomerular immune deposits of most of these patients consisted of IgG, and only 2 patients showed IgA-type monoclonal deposition. To the best of our knowledge, our present patient is the first patient to date showing MGRS presenting as MN with IgM-type monoclonal deposition. Although rare, there have been a number of reported cases of patients with MN showing dominant IgM deposition (16, 17). Among them, there has been a case of a patient with IgM lambda-type MN accompanied with Waldenström macroglobulinemia (WM), who was treated effectively with rituximab and fludarabine. Consistent with these previous reports, steroid therapy was ineffective, but rituximab treatment improved both the hematological and renal abnormalities of our present patient.

Interestingly, the IF staining pattern was clearly different in the second renal biopsy of our patient compared with the first. In the second biopsy, glomerular IgM staining appeared weaker, whereas IgG staining was augmented, in which IgG2 was dominant with weaker deposition of IgG4. In addition, both lambda and kappa light-chain deposition were almost equally observed. The serum anti-PLA_2_R antibody level, as well as immunostaining for PLA_2_R and NELL-1, a recently identified antigen observed in a distinct type of MN (18) were negative, whereas that for THSD7A showed diffuse and global granular-positive staining on the glomerular capillary walls. It was reported that anti-THSD7A antibodies were predominantly of the IgG4 subclass (2), which might explain the appearance of the IgG4 subclass in the second renal biopsy of the present patient. On the other hand, focal and segmental granular-positive immunostaining for THSD7A was already observed in the first renal biopsy tissue. In this regard, we considered the possibility that specific immune responses occurring during the progression of MN in this patient induced the exposure of THSD7A epitopes, resulting in the production of THSD7A autoantibodies. Consistent with this scenario, Tominaga et al. (19) recently reported two cases of patients with MN-lesions, in which both myeloperoxidase and PLA_2_R were detected in the glomerular subepithelial deposits, suggesting that a specific inflammatory environment led to the abnormal exposure of PLA_2_R epitopes. Another possibility is that IgM lambda antibodies, which were observed to be deposited in the first renal biopsy tissue, were already bound to THSD7A, and IgG antibodies to THSD7A developed thereafter by Ig class switching. Although THSD7A was reported to be expressed in the malignant tumor tissue of a patient with THSD7A-associated MN (5), immunoperoxidase staining for THSD7A was negative in the bone marrow tissue of the present patient (Supplementary Figure 3).

We assumed that glomerular IgM deposition was not decreased in the second biopsy, but may have been difficult to detect owing to the deposition of polyclonal IgG, thereby interfering the immuno-reactivity of anti-IgM antibody. IgG subclass staining showed dominant IgG2 deposition in the second renal biopsy tissue. Although several new antigens in MN have recently been proposed (3), no corresponding antibodies predominantly of the IgG2 subclass have been identified to date. Thus, polyclonal IgG antibodies that react with monoclonal IgM, which are deposited on the glomerular capillary walls, may be produced via autoimmune mechanisms in this patient. Indeed, immunoperoxidase staining on FFPE sections of the second renal biopsy after antigen retrieval showed strong IgM deposition on the glomerular capillary walls (Figure 2G). Strong IgM deposition along the capillary walls was also shown by IF staining on a fresh frozen tissue section of the second renal biopsy after pretreatment with acidic buffer for antigen retrieval (Supplementary Figure 4).

Regarding treatment regimens for MGRS patients, targeting the causative B-cell clone is considered to be a reasonable therapeutic strategy (8). Indeed, in our patient, improvement of her nephrotic syndrome as well as an adequate hematological response was achieved not by ARB or steroid therapy but by rituximab treatment. It should be noted, however, that favorable therapeutic effects of rituximab have been widely reported in the treatment of nephrotic syndrome.

In conclusion, we reported a case of a patient with MGRS presenting as MN with monoclonal IgM lambda deposits accompanied by IgM lambda monoclonal gammopathy, in which improvement of renal and hematological abnormalities was achieved by rituximab treatment. Histological observation of repeated biopsies demonstrated that glomerular monoclonal IgM staining seen in an early disease phase appeared to be weakened by the deposition of polyclonal IgG in a late disease phase, and that the exposure of THSD7A epitopes and the resultant production of THSD7A autoantibodies might be involved in disease progression. This suggested that a diagnosis of MN with monoclonal Ig deposits would be difficult if histological analyses were performed only at a late phase of the disease and in a routine manner. Further accumulation and analyses of patients with MN with monoclonal Ig deposits are therefore necessary to establish an appropriate diagnosis and treatment methods.

## Data Availability Statement

The original contributions presented in the study are included in the article/Supplementary Material, further inquiries can be directed to the corresponding author/s.

## Ethics Statement

Ethical review and approval was not required for the study on human participants in accordance with the local legislation and institutional requirements. The patients/participants provided their written informed consent to participate in this study. Written informed consent was obtained from the individual(s) for the publication of any potentially identifiable images or data included in this article.

## Patient's Perspective

The patient has remained in a stable and improved condition, as the peripheral edema in her lower legs, as well as other presenting symptoms, resolved after rituximab treatment.

## Author Contributions

GH and TU: writing the manuscript draft. TO: critical manuscript revision. GH, AK, and MY: clinical care of the patient. TU, KS, YN, TT, KN, and TO: histological evaluation. All authors contributed to the article and approved the submitted version.

## Conflict of Interest

The authors declare that the research was conducted in the absence of any commercial or financial relationships that could be construed as a potential conflict of interest.

## Supplementary Material

Supplementary Figure 1 Immunofluorescence staining of formalin-fixed, paraffin-embedded tissue (FFPE) sections showed granular deposition of immunoglobulin (Ig)M **(A)** and lambda chain **(B)** on the glomerular capillary walls, whereas there was no deposition of IgG **(C)**, IgA **(D)**, or kappa chain **(E)**.

Supplementary Figure 2 Immunofluorescence staining for the phospholipase A_2_ receptor **(A)** and immunoperoxidase staining for neural epidermal growth factor-like 1 protein **(B)** on FFPE sections of the second renal biopsy tissue. Immunoperoxidase staining for amyloid P on FFPE sections of the first renal biopsy tissue **(C)** and the second renal biopsy tissue **(D)**.

Supplementary Figure 3 Immunoperoxidase staining for thrombospondin-type-1-domain-containing-7A (THSD7A) on FFPE sections of renal biopsy tissues and bone marrow tissue. The staining was weak, focal, segmental, and granular on the capillary walls of the first renal biopsy tissue **(A)**, whereas the staining was strong, diffuse, global, and granular on the capillary walls of the second renal biopsy tissue **(B)**. Bone marrow cells, including neoplastic plasma cells, were negative for THSD7A by immunoperoxidase staining **(C)**.

Supplementary Figure 4 Immunofluorescence staining for IgM on fresh frozen tissue sections of the second renal biopsy. Acetone-fixed cryostat sections of fresh-frozen renal biopsy tissues, with or without incubation with acidic buffer consisting of 0.1 M potassium chloride and 0.1 M hydrochloric acid for 30 min at room temperature, were immunohistochemically stained for IgM. Note that preincubation with the acidic buffer demonstrated extensive IgM deposition along the capillary walls (**A**: without acidic buffer, **B**: with acidic buffer).
