# Supplementary figures and images for: Membranous Nephropathy With Monoclonal IgM Lambda Deposits in a Patient With IgM Monoclonal Gammopathy: A Case Report
Source: Front Med (Lausanne). 2021 May 25;8:608741. doi: 10.3389/fmed.2021.608741 (PMC8185035; doi:10.3389/fmed.2021.608741)

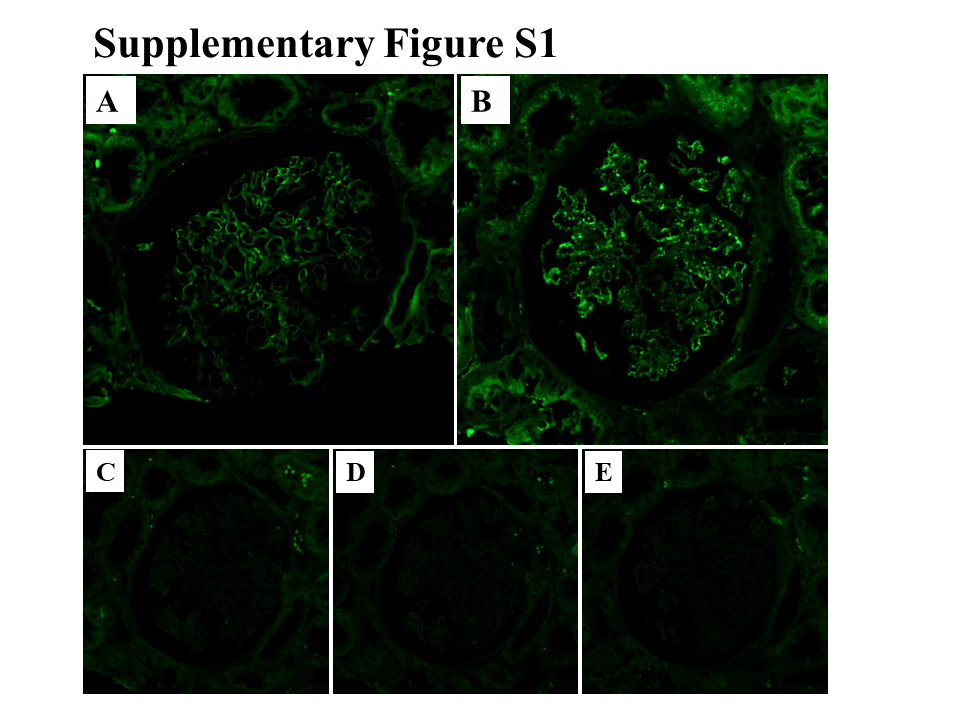

Supplement: Supplementary Figure 1 — Immunofluorescence staining of formalin-fixed, paraffin-embedded tissue (FFPE) sections showed granular deposition of immunoglobulin (Ig)M (A) and lambda chain (B) on the glomerular capillary walls, whereas there was no deposition of IgG (C), IgA (D), or kappa chain (E). [file Image_1.TIF]

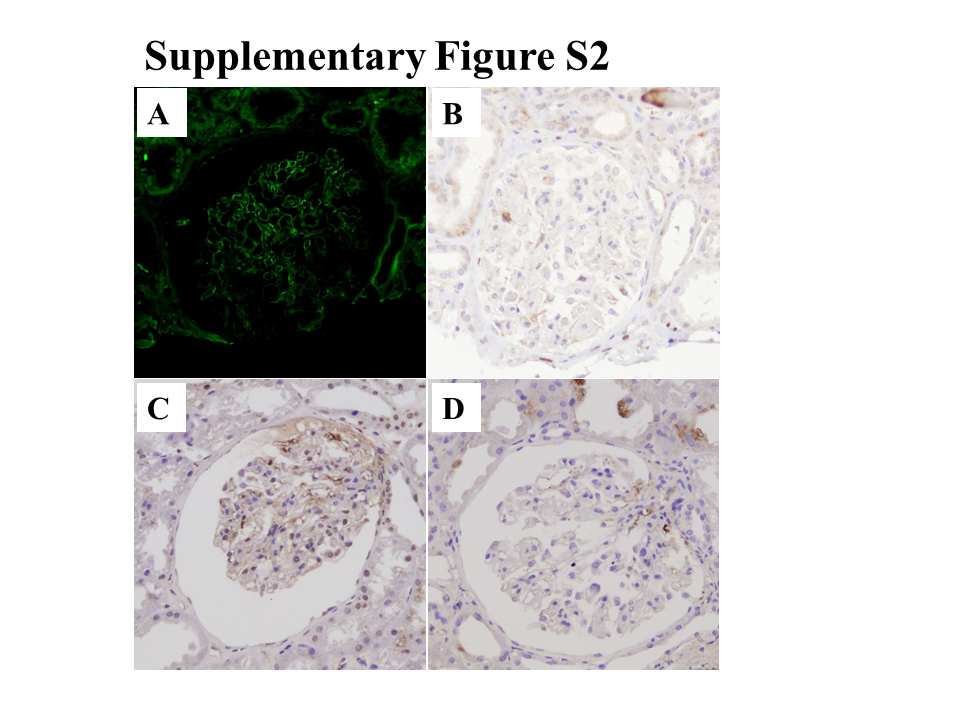

Supplement: Supplementary Figure 2 — Immunofluorescence staining for the phospholipase A2 receptor (A) and immunoperoxidase staining for neural epidermal growth factor-like 1 protein (B) on FFPE sections of the second renal biopsy tissue. Immunoperoxidase staining for amyloid P on FFPE sections of the first renal biopsy tissue (C) and the second renal biopsy tissue (D). [file Image_2.TIF]

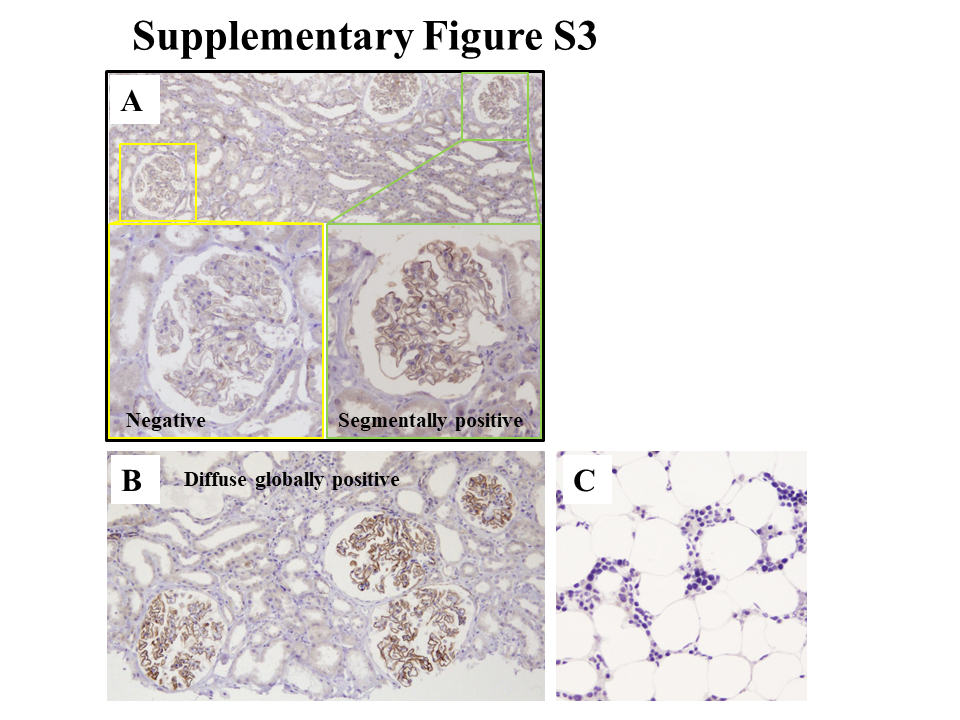

Supplement: Supplementary Figure 3 — Immunoperoxidase staining for thrombospondin-type-1-domain-containing-7A (THSD7A) on FFPE sections of renal biopsy tissues and bone marrow tissue. The staining was weak, focal, segmental, and granular on the capillary walls of the first renal biopsy tissue (A), whereas the staining was strong, diffuse, global, and granular on the capillary walls of the second renal biopsy tissue (B). Bone marrow cells, including neoplastic plasma cells, were negative for THSD7A by immunoperoxidase staining (C). [file Image_3.TIF]

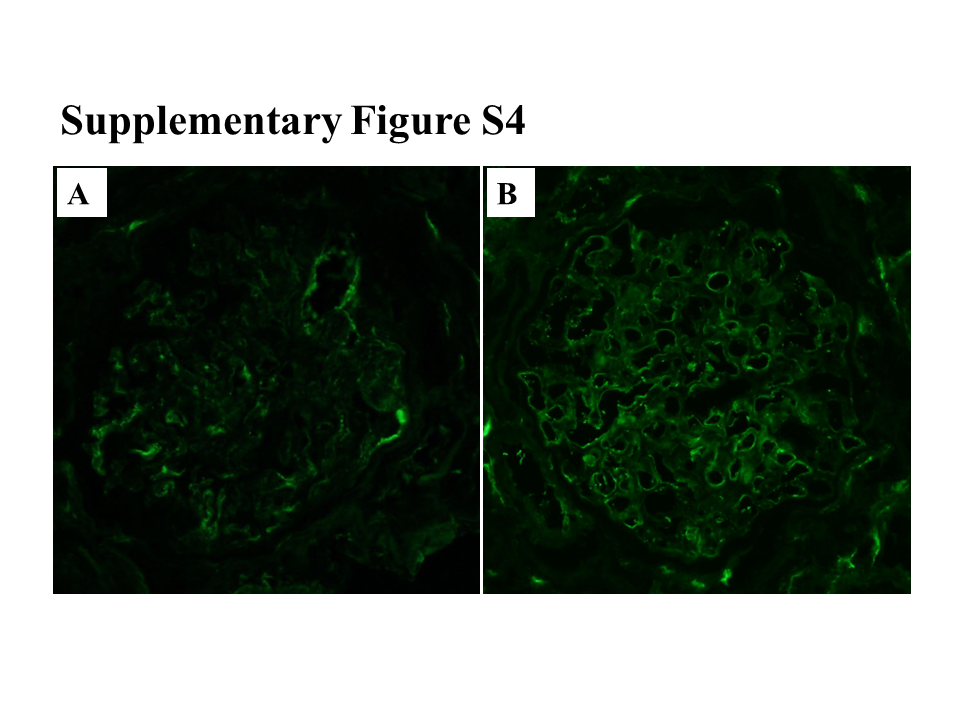

Supplement: Supplementary Figure 4 — Immunofluorescence staining for IgM on fresh frozen tissue sections of the second renal biopsy. Acetone-fixed cryostat sections of fresh-frozen renal biopsy tissues, with or without incubation with acidic buffer consisting of 0.1 M potassium chloride and 0.1 M hydrochloric acid for 30 min at room temperature, were immunohistochemically stained for IgM. Note that preincubation with the acidic buffer demonstrated extensive IgM deposition along the capillary walls (A: without acidic buffer, B: with acidic buffer). [file Image_4.TIF]
